# *Lepidium meyenii *(Maca) increases litter size in normal adult female mice

**DOI:** 10.1186/1477-7827-3-16

**Published:** 2005-05-03

**Authors:** Ana C Ruiz-Luna, Stephanie Salazar, Norma J Aspajo, Julio Rubio, Manuel Gasco, Gustavo F Gonzales

**Affiliations:** 1 Instituto de Investigaciones de la Altura, Universidad Peruana Cayetano Heredia, Lima, Perú; 2 Department of Biological and Physiological Sciences, Faculty of Sciences and Philosophy, Universidad Peruana Cayetano Heredia, Lima, Perú

## Abstract

**Background:**

*Lepidium meyenii*, known as Maca, grows exclusively in the Peruvian Andes over 4000 m altitude. It has been used traditionally to increase fertility. Previous scientific studies have demonstrated that Maca increases spermatogenesis and epididymal sperm count. The present study was aimed to investigate the effects of Maca on several fertility parameters of female mice at reproductive age.

**Methods:**

Adult female Balb/C mice were divided at random into three main groups: i) Reproductive indexes group, ii) Implantation sites group and iii) Assessment of uterine weight in ovariectomized mice. Animals received an aqueous extract of lyophilized Yellow Maca (1 g/Kg BW) or vehicle orally as treatment. In the fertility indexes study, animals received the treatment before, during and after gestation. The fertility index, gestation index, post-natal viability index, weaning viability index and sex ratio were calculated. Sexual maturation was evaluated in the female pups by the vaginal opening (VO) day. In the implantation study, females were checked for implantation sites at gestation day 7 and the embryos were counted. In ovariectomized mice, the uterine weight was recorded at the end of treatment.

**Results:**

Implantation sites were similar in mice treated with Maca and in controls. All reproductive indexes were similar in both groups of treatment. The number of pups per dam at birth and at postnatal day 4 was significantly higher in the group treated with Maca. VO day occurred earlier as litter size was smaller. Maca did not affect VO day. In ovariectomized mice, the treatment with Maca increased significantly the uterine weights in comparison to their respective control group.

**Conclusion:**

Administration of aqueous extract of Yellow Maca to adult female mice increases the litter size. Moreover, this treatment increases the uterine weight in ovariectomized animals. Our study confirms for the first time some of the traditional uses of Maca to enhance female fertility.

## Background

Maca, is a cruciferous plant that grows exclusively between 4,000 and 4,500 m altitude in the Central Peruvian Andes [1]. The hypocotyl-root axis is the edible part of the plant, which is dried and can be stored in this way for years. This hypocotyl has been used for centuries as nutrient and by its fertility-enhancer property [2-4].

Scientific studies in male rats and mice have demonstrated that oral administration of aqueous or ethanolic extracts of Maca hypocotyls improved spermatogenesis [1,5-7]. Moreover, an improvement in sperm count was observed in men after four months of treatment [8].

Studies reported in the peer-reviewed scientific literature about the effects of this native plant in the female reproductive system are scarce. The only scientific evidence reported showed that Maca did not modify the rate of implantation. In that study, Maca was mixed in the food for 30 days [9]. For such reason, the dose administered was not controlled.

The present study was aimed to investigate the effects of an aqueous extract of Maca in female mice at reproductive age through several female reproductive indexes, uterine weight measurements, number of implanted embryos and vaginal opening (VO) day of the female pups with a fixed dose of Maca administered to the dams.

## Methods

### Animals

Three-month-old virgin female mice of the Balb/C strain obtained from the Animal House at the National Institute of Health (NIH) in Peru were used for this study. Mice were divided at random into three main groups: i) The first group was used to assess reproductive indexes; ii) the second group was aimed to study implantation sites and iii) the last group was aimed to evaluate the effect of Maca on uterine weight in ovariectomized mice.

Each of the three main groups was divided into two subgroups: the control group, which received 0.5 ml distilled water (vehicle) and the treated group, which received lyophilized aqueous extract of Yellow Maca orally by gavage. This study was conducted in accordance with Universidad Peruana Cayetano Heredia Guidelines and Ethics on Animal Experimentation.

### Preparation of the Aqueous Extract of Maca

Dried Maca was provided by Santa Natura Company. The ecotype used for this study was Yellow Maca. Dried Maca hypocotyls were crushed in a mill, diluted 1:3 w/v in distilled water and boiled for approximately 2 hours. Afterwards, they were filtered and let at room temperature for cooling. Aqueous extract was freezed under -70°C for three days and then lyophilized. One gram of lyophilized Maca is equivalent to 2.19 g of dried Maca hypocotyls. Lyophilized Maca was diluted in distilled water to obtain a dose of 1 g/kg BW. This dose has been proved to be optimal in a dose-dependent study [10].

### Implantation sites

Female mice were treated with lyophilized aqueous extract of Yellow Maca (1 g/Kg) or distilled water (vehicle) by oral route for 22–28 days (15 days prior mating and during the first seven days of gestation period). At day 15 of treatment with Maca or vehicle, each female mouse was mated with a single male of proven fertility. The presence of a vaginal plug was considered the first day of pregnancy. The females were killed by cervical dislocation at day 7 of gestation and the uterus was checked for implantation sites. The embryos were counted and weighed.

### Reproductive indexes

Female mice were administered orally by gavage with distilled water (vehicle) or lyophilized aqueous extract of Maca (1 g/kg body weight) for 15 days prior to mating, during the whole period of gestation and 21 days after birth (lactation period). Body weight was recorded daily.

At day 15 of treatment with vehicle or Maca, each female mouse was housed independently of the estrous cycle with a sexually mature untreated male of proven fertility. The presence of a copulation plug in the vagina was regarded as successful copulation and was considered the first day of pregnancy. For pregnancy confirmation, mice were examined by vaginal smear to determine the presence of diestrus stage cells during a 7 day-period after the finding of a vaginal plug.

Upon delivery, the number of pups was counted. Both groups of dams continued their respective treatments during the whole lactation period (21 days). The number of pups alive at postnatal day 4 and 21, the VO day of the female pups and the sex ratio of both groups were also determined (male fraction).

The following reproductive indexes were calculated [11]: fertility index, defined as No pregnant females/No females with successful copulation × 100; gestation index, defined as No of females with alive pups/ No of pregnant females × 100; post-natal viability index, defined as No of pups alive on day 4 / No of alive pups × 100; and weaning viability index, defined as No of pups alive at day 21/ No of pups alive at day 4 × 100.

### Ovariectomized mice

In order to assess a possible estrogenic role of Maca on uterine weight, 3 month-old mice were ovariectomized. Animals were shaved in the dorsal surgery area and anesthetized with ketamide (40 mg/kg body weight i.p). A dorsal incision was performed and the ovaries were exposed and removed. Three months after surgery, 15 animals were exposed orally by gavage to distilled water (n = 8; 0.5 ml distilled water) or Yellow Maca (n = 7; 0.5 g/kg body weight) for a 42-day period. At the end of the treatment, animals were sacrificed by cervical dislocation. Body and uterine wet weights were recorded.

### Statistical analysis

Prior to the analysis, normality of the data was determined using the one sample Kolmogorov-Smirnov Test. Body weight, implantation sites, gestation length and litter size variables were analyzed using Student's t test. Multivariate analysis was used to analyze the influence of the Maca treatment on the number of pups and on the VO day. Reproductive indexes were analyzed using the Paired Proportion Test. Data are presented as mean ± S.E.M. Data without normal distribution, such as the vaginal opening day and the uterine weight were analyzed using the Mann Whitney U Test. Groups were considered significantly different if P < 0.05. All calculations and statistical analysis were generated in STATA v 8.0 (Stata Corporation, College Station, TX, USA).

## Results

### Indexes

In both Maca treated and control groups, the fertility index, the gestation index, the post-natal viability index and the weaning viability index were similar between the control and the Maca treated group (Table 1).

**Table 1:** Reproductive indexes in female mice receiving Maca (1 g/kg) distilled water

Indexes (%)	Distilled water (n = 12)	Maca (n = 11)
Fertility index^a^	100.0	100.0
Gestation index^a^	100.0	100.0
Post-natal viability index^a^	94.3	97.1
Weaning viability index^a^	96.3	96.0

^a ^No statistical difference was found between both groups (P > 0.05).

### Body Weight

In both the implantation study and the reproductive indexes study, the body weight did not differ significantly between control and Maca-treated groups. In the implantation study, at day 1, body weights were 34.68 ± 1.12 g in the control group and 34.13 ± 0.62 g in the Maca-treated group. After 17 days of treatment with lyophilized aqueous extract of Yellow Maca, body weight was similar to that observed in the control group (control group, 33.43 ± 1.18 g vs. Maca group, 34.98 ± 0.72 g). Body weight change between day 1 and day 17 of treatment was similar in the Maca treated animals and in controls (0.85 ± 0.30 g vs. -1.25 ± 1.17 g, respectively).

In the reproductive indexes study, body weight at day 1 of treatment with distilled water (control group) was 29.4 ± 0.57 g and with Maca was 29.94 ± 0.82 g. At day 15 of treatment, body weight of the control group was 30.77 ± 0.61 g, while the group treated with Maca showed a body weight of 32.07 ± 1.27 g. No differences between groups of treatment at day 1 or day 15 were observed.

### Implantation sites

The implantation sites were similar in the Maca treated group and the controls (11.56 ± 2.13 vs. 9.67 ± 2.42, respectively).

### Gestation length

The gestation length was similar in the Maca-treated group and the control group (19.88 ± 0.11 days and 19.92 ± 0.08 days, respectively).

### Pups/ Litter size

Mean number of pups at birth and at postnatal day 4 in the Maca treated group was higher than their respective controls (P < 0.05) (Table 2). Multivariate analysis showed that treatment with Maca increase number of pups (3.18 ± 1.04; Coefficient of regression ± SE; P < 0.05).

**Table 2:** Effects of Maca (1 g/kg BW) on litter size, vaginal opening day and sex ratio.

	Distilled water	Maca
Litter size	(n = 12)	(n = 10)
Day 1 (birth)	7.25 ± 1.14	10.40 ± 1.32^a^
Day 4	6.83 ± 1.28	10.10 ± 1.30^a^
Day 21	6.58 ± 1.33	9.70 ± 1.30
Vaginal opening day (days)	(n = 37)	(n = 52)
Litter < 7 pups	26.1 ± 0.48	23.2 ± 0.14^‡‡^
Litter ≥ 7 pups	27.5 ± 0.27*	28.40 ± 0.46***
Sex ratio (male fraction)	0.54	0.50

^a^P < 0.05 with respect to distilled water group.

*P < 0.05, ***P < 0.001 with respect to values in the group with litter size below 7. ^‡‡^P < 0.01 with respect to distilled water group.

### Sex ratio

The fraction of male pups in the control group and in the treatment group were not statistically different between Control and Maca group (0.54 and 0.50, respectively) (Table 2).

### Vaginal opening

In order to assess the effect of Maca on female sexual maturation, the vaginal opening (VO) day was evaluated. During postnatal development of pups, it was noticed that those belonging to a bigger litter size developed and grew slower in size and weight in comparison to the smaller litters (data not shown). Statistical analysis showed that the VO day was indeed influenced by the litter size. Therefore, the litters were divided in two subgroups: litters with equal or more than seven pups and litters with less than seven pups. Litters with <7 pups showed lower VO day than litters with litters ≥ 7 (P < 0.01). Maca treated group produced an earlier VO day in litters with less than 7 pups than in the controls (P < 0.01). In litters with ≥ 7 pups, the VO day was similar in the Maca treated group and in the control group. The multivariate analysis showed that litter size but not treatment was related to VO day (R^2 ^= 0.37; P < 0.01).

#### Ovariectomized mice

Oral administration of lyophilized aqueous extract of Yellow Maca for 42 days significantly increased uterine wet weight (65.56 ± 16.62 mg; mean ± SEM) when compared to their respective controls (29.98 ± 3.05 mg) (P < 0.05).

## Discussion

Maca has been described to improve fertility since many centuries ago [3]. However, scientific evidences in peer reviewed journals were available since 2000 [5-8,12-14]. The studies demonstrated that Maca increases male sexual behavior [11-13] and increases sperm production [8].

In relation to female reproduction, there is only one study demonstrating that Maca did not increase implantation rate in mice [9]. We have demonstrated in the present study that implantation rate was not increased in mice after oral administration of 1 g/Kg BW of a lyophilized aqueous extract of Maca. However, interestingly, female mice that received Maca delivered more pups than the control group. This suggests that Maca may have a protective effect on the number of resorptions, having a less occurrence of them between day 7 of pregnancy and the subsequent days of pregnancy in the Maca treated group. Supplementation of Maca in diets improved growth rates and survival of rainbow trout *Oncorhynchus mykiss *(Walbaum) alevins and juveniles [15]. Some possible mechanisms through which Maca may act include increased uterine receptivity, altered immune functions and effects on vascular system [16]. It is known that functional lymphocytic progesterone binding sites are needed for the maintenance of normal pregnancy, and that progesterone-mediated immunosuppression is needed for the maintenance of normal gestation [17].

Litter size was higher at birth and at postnatal day 4 in the groups treated with Maca than in the controls. This effect seems to be due to a reduction in embryo resorption, since number of implantation sites were similar in mice treated with Maca than in controls. This effect seems not to be due to any estrogenic activity of Maca, since exposure to estradiol-17 beta or the proestrogen methoxychlor reduced litter size [18].

Sex ratio was not affected after treatment with Maca. In fish, the period of sex differentiation is the most sensitive to possible action of phytochemicals with steroid activity. However, there was no significant difference in sex ratios between the control and Maca treated group of rainbow trout. P-nonyphenol, an environmental toxicant with estrogenic properties did not affect the sex ratio of live pups [19]. All of these suggest that main effects of Maca were not due to increased estrogenic activity.

Sexual maturation in the litters was evaluated by the vaginal opening day. The pups were divided in two groups (< 7 pups and ≥ 7 pups). Fraternity size has a positive linear effect on age at vaginal opening. Lower litter size was related to early age at vaginal opening day. This has been observed previously [20]. This difference seems to be due to differences in dietary energy density [21]. When comparing both sub-groups with their respective controls, we observed that Maca had not effect on the VO day. Dietary phytoestrogens has been shown to accelerate the time of vaginal opening in immature CD-1 mice [22]. Since Maca does not affect the VO day, it is suggested that phytoestrogens present in Maca has little impact on reproductive parameters assessed in the present study.

Maca contains sterols, such as campesterols (27.3%), ergosterol (13.6%), brassicasterol (9.1%) Δ^7,22^-ergostadienol (4.5%) and sitosterol (45.5 %) [23], the latter having phytoestrogen activity. It has been suggested that β-sitosterol could cross the placenta [24]. In humans, it has been demonstrated that sitosterol can be excreted in milk and therefore, enter the neonate [25]. Maca chemical composition includes sitosterol, this compound would exert its effect on pups.

Treatment with Maca also increases male sexual behavior [12-14] and sperm production in mice and rats [7]. It is probable that main effects of Maca increasing uterine weight and litter size were not due to an estrogenic effect but to a progestin-like one, since Maca chemical composition include other sterols besides phytoestrogen sitosterol. Progesterone would certainly affect both, uterine weight via progesterone receptors, as well as prevent abortion. In mice and rats, progesterone is much more important in maintaining pregnancy than estrogen [16,17]. Moreover, physiological level of progesterone acts in conjunction with androgens to facilitate copulatory behavior in male rats and mice [26,27]. In addition, one of the most potent phytoestrogenic substance -6-(1,1-dimethylallyl) naringenin (6-DMAN)- did not have any effect on uterine wet weight in ovariectomized rats [28]. Our study demonstrates that Maca increases uterine weight. It is suggested that this activity may be produced by a progestin-like effect. However, more studies need to be performed to clarify this proposed mechanism.

## Conclusion

To sum up, our results suggest that lyophilized aqueous extract of Yellow Maca increase the number of pups in normal adult female mice.

## Authors' contributions

AR conceived of the study, participated in its design, coordination, execution, analysis and interpretation of the data, and drafted the manuscript.

SS participated in the design, coordination and execution of the experiment, analysis of the and interpretation of the data, and helped draft the manuscript of the study.

NA participated in the development of the experiment and revised the draft critically.

JR participated in the development of the experiment, carried out the analysis and interpretation of the data, and revised the draft critically.

MG participated in the development of the experiment.

GG participated in the analysis and interpretation of the data, helped draft the manuscript, revised it critically and gave the final approval of the version.
